# Steroid Premedication and Monoclonal Antibody Therapy: Should We Reconsider?

**DOI:** 10.1007/s11864-023-01170-4

**Published:** 2024-01-03

**Authors:** Emma-Anne Karlsen, Euan Walpole, Fiona Simpson

**Affiliations:** 1 Frazer Institute, The University of Queensland, Brisbane, Australia; 2 Department of General Surgery, Mater Hospital Brisbane, Brisbane, Australia; 3 School of Medicine, The University of Queensland, Brisbane, Australia; 4 Simpson Laboratory - Frazer Institute, The University of Queensland, 37 Kent Street, Woolloongabba, QLD 4102 Australia; 5 Division of Cancer Services, Princess Alexandra Hospital, Brisbane, Australia

**Keywords:** Monoclonal antibodies, Antibody-dependent cell-mediated cytotoxicity (ADCC), Natural killer (NK) cells, Cetuximab, Trastuzumab, Monoclonal antibody premedication, Steroid

## Abstract

Monoclonal antibody (mAb) therapy is now considered a main component of cancer therapy in Australia. Although traditionally thought of as pure signalling inhibitors, a large proponent of these medications function through antibody-dependent cell-mediated cytotoxicity (ADCC). Currently, most protocols and institutional guidelines for ADCC-mediated mAbs promote the use of corticosteroids as premedication: this is implemented to reduce infusion-related reactions (IRRs) and antiemesis prophylaxis and combat concurrently administered chemotherapy-related syndromes. Concerningly, the inhibitory effects of ADCC by corticosteroids are well documented; henceforth, it is possible the current standard of care is misaligned to the literature surrounding ADCC. Subsequently, clinicians’ decisions to act in contrast to this literature may be reducing the efficacy of mAbs. The literature suggests that the redundant use of corticosteroids should be cautioned against when used in conjunction with ADCC-mediated mAbs—this is due to the consequent reduction in anti-tumour activity. Owing to the fact IRRs typically occur upon initial infusion, the authors advocate for individual clinicians and institutional protocols to considering augmenting their practice to corticosteroid premedication at the first dose only, unless clinically indicated. Additionally, product information (PI) and consumer medicine information (CMI) documents distributed by Australian and international regulatory agencies should consider disclosing the risk of concurrent steroids with these medications. Moreover, the authors suggest considering alternative medications for the management of side effects.

## Introduction

Monoclonal antibody (mAb) therapy has revolutionised the anti-cancer landscape by offering targeted therapy that reduces the severe side effects often associated with conventional systemic therapy. In Australia, mAbs and antibody–drug conjugates have become a cornerstone of standard of care treatment for several solid and haematological cancers. Although conventionally thought of as signalling inhibitors, a significant portion of mAbs have since been demonstrated to function largely through their engagement with Fc receptors to activate innate immune effector cells to mediate antibody-dependent cell-mediated cytotoxicity (ADCC).

Australian and international guidelines incorporate steroids as an integral part of mAb premedication as emesis prophylaxis and to combat infusion-related reactions (IRRs)—most of which occur with the first dose. However, many institutions continue steroid administration throughout the full course of mAb treatment. The following will discuss the risks of continued steroid administration due to the resultant reduction in ADCC, the primary mechanism of action of a significant number of mAbs utilised by medical oncologists.

## Antibody-dependent cell-mediated cytotoxicity

Antibodies directed against tumour cell antigens can lead to tumour cell death via direct and indirect mechanisms. Direct mechanisms include blocking growth factor receptor signalling; direct transmembrane signalling; and acting as vectors for toxic payloads (e.g. radioisotopes) [1]. Indirect mechanisms require engagement with the host immune system and include complement-mediated cytotoxicity; antibody-dependent cellular phagocytosis; and ADCC.

**Fig. 1:**
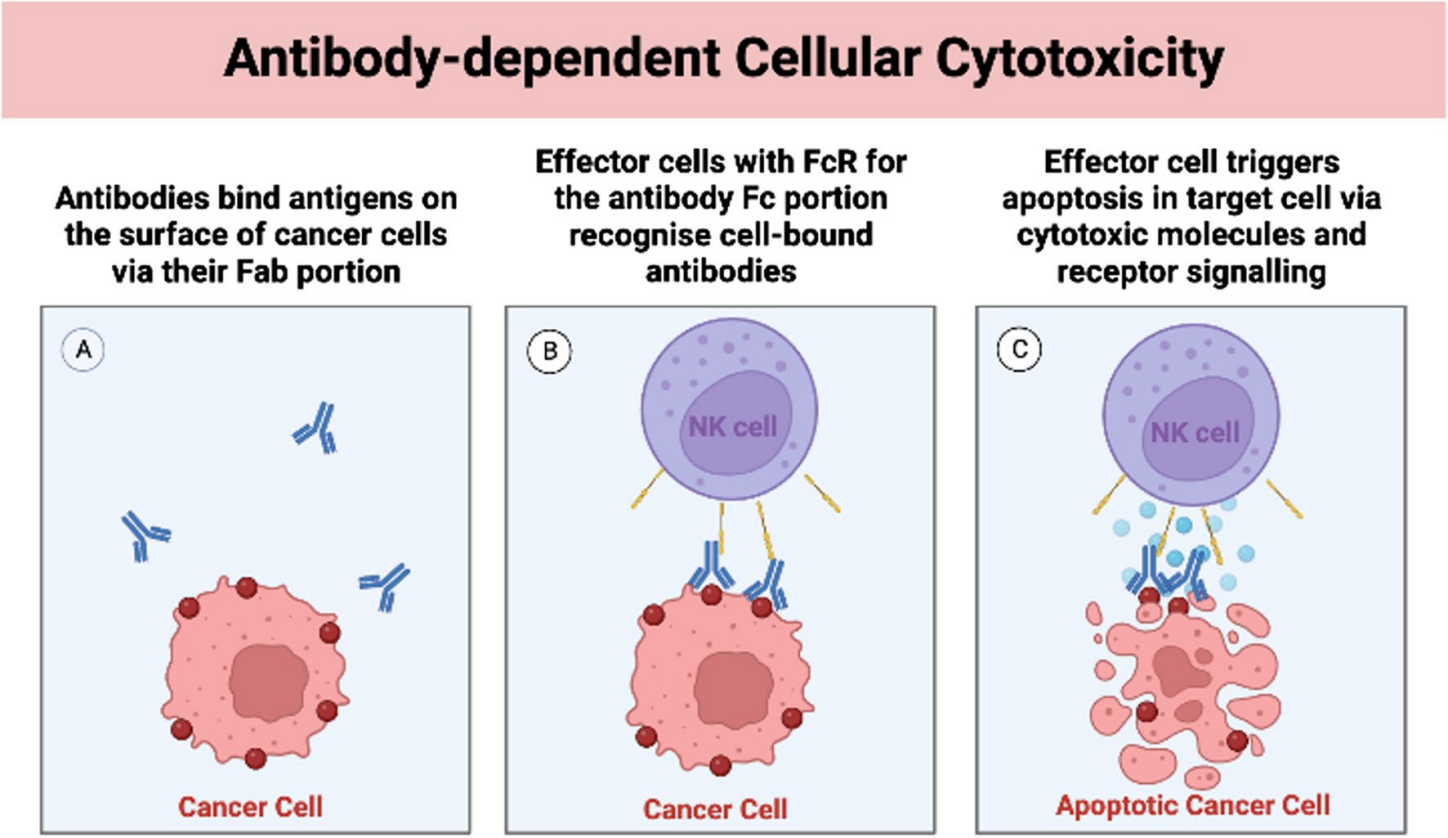
Antibody-dependent cellular cytotoxicity. Created with BioRender.com.

ADCC plays a particularly important role in the efficacy of IgG1 antibodies in cancer therapy [2, 3]. As depicted in Fig. 1, ADCC is a specific process by which antibodies bind to a cancer cell surface antigen by their fragment antigen-binding (Fab) portion [4, 5]. Following this binding, the fragment crystalline (Fc) portion of the antibody interacts with the Fc receptor (FcR) on the surface of an effector cell: such as a natural killer (NK) cell or macrophage which consequently initiates ADCC. Subsequent effector cell–induced apoptosis is achieved via mechanisms including cytotoxic granule release, Fas signalling and reactive oxygen species.

The list of anti-neoplastic mAbs and ADCs currently approved by Australian Pharmaceutical Benefits Scheme (PBS) was reviewed for those known to be mediated by ADCC and is collated in Table 1. Developed in 1997, rituximab was the first monoclonal antibody approved for the treatment of cancer. Rituximab is a CD-20 targeting mAb whose Fc portion induces lymphoma cell lysis through ADCC [6, 7]. Obinutuzumab, a new generation of anti-CD20 antibody, was designed in an attempt to overcome postulated mechanisms of resistance, however still retains ADCC as a major mechanism of action [8].

**Table 1:** Pharmaceutical Benefits Scheme (PBS)-approved ADCC-mediated therapeutic monoclonal antibodies and antibody–drug conjugates

Antibody (company)	Type	Target	Tumour type
Obinutuzumab (Roche Products)	Humanised IgG1	CD20	Chronic lymphocytic leukaemia (CLL), follicular lymphoma
Rituximab (Pfizer Australia)	Chimeric IgG1	CD20	Non-Hodgkin’s lymphoma, CLL
Daratumumab (Janssen-Cilag)	Humanised IgG1	CD38	Light-chain amyloidosis
Pertuzumab (Roche Products)	Humanised IgG1	HER2	HER2-positive breast cancer
Trastuzumab (Alphapharm)	Humanised IgG1	HER2	HER2-positive breast cancer, gastric cancer
Trastuzumab emtansine (Roche)	Humanised IgG1 covalently linked to DM1, a microtubule polymerisation inhibitor	HER2	HER2-positive breast cancer
Trastuzumab deruxtecan (AstraZeneca)	Humanised IgG1 covalently linked to a topoisomerase I inhibitor	HER2	HER2-positive breast cancer
Cetuximab (Merck Serono)	Chimeric IgG1	EGFR	EGFR-expressing, KRAS wild-type colorectal cancer (CRC), head and neck squamous cell carcinoma (SCC)
Panitumumab (Amgen)	Humanised IgG2	EGFR	EGFR-expressing, KRAS wild-type CRC
Avelumab (Merck)	Human IgG1	PD-1/PDL-1	Merkel cell carcinoma
Blinatumomab (Amgen)	Bi-specific T cell engager (BiTE)	CD3-CD19	B cell precursor acute lymphoblastic leukaemia
Elotuzumab (Bristol-Myers Squibb)	Humanised IgG1	SMALF7 pathway	Multiple myeloma
Ipilimumab (Bristol-Myers Squibb)	Human IgG1	CTLA-4	Melanoma

Cetuximab is a chimeric mouse-human antibody targeted against the extracellular domain of EGFR. Cetuximabs’ primary use is via inhibition of ligand-dependent receptor activation and inhibition of downstream cell growth pathways. However, owing to the IgG1 backbone, cetuximab has proven to demonstrate significant ADCC activity in several cancers [9–13, 14•]. A growing body of evidence suggests that the cytotoxic ability of cetuximab is largely dependent on NK cells [14•]. This is due to their direct ADCC activity as well as the ability to trigger antigen-specific T cell immunity [15].

Similar to cetuximab, the anti-HER2 mAb trastuzumab also induces ADCC mediated by NK cells and has been proven to play an important role in tumour clearance [14•, 16, 17]. With reference to Fig. 1, the Fc region of trastuzumab bound to their target on cancer cells can bind Fcγ receptors (FcγR) on NK cells—triggering ADCC via the release of granzymes and perforin [4, 18, 19]. The importance of ADCC on tumour clearance is highlighted by the observation of severely attenuated trastuzumab activity in FcγR-deficient mice [16].

Avelumab binds to programmed death ligand (PD-L1), inhibiting PD1-PDL1 interaction. In addition to its checkpoint inhibition, avelumab also stimulates ADCC and antibody-dependent cellular phagocytosis [20].

## Steroid premedication for therapeutic monoclonal antibodies

The eviQ protocols were used as a representative sample for current standard of care cancer treatment protocols in Australia [21]. Upon revision of the proposed premedication regimens for the mAbs noted in Table 1, corticosteroids form an integral and recurring portion that exceeds the first cycle. In the neoadjuvant treatment of breast cancer, for docetaxel, pertuzumab and trastuzumab, 8 mg of oral dexamethasone is protocolised the day prior to chemotherapy, day 1 and day 2 for cycles 1–4 [22]. Similarly, for metastatic breast cancer, it is recommended that 8 mg of oral dexamethasone be administered on days 1–4 of all cycles of trastuzumab deruxtecan [23]. This recommendation is paralleled in the European Society for Medical Oncology journal which recommends dexamethasone premedication for all cycles as nausea and vomiting prophylaxis [24].

A recent article published in the British Journal of Clinical Pharmacology investigated all patients treated with trastuzumab at a single site for a period of 4 years and found that 3.4% of infusions were associated with an IRR, with 91.4% of these reactions occurring during the first dose [25]. This paper concluded that dexamethasone premedication was effective in reducing trastuzumab-induced IRR [25], however explicitly noted that they would not recommend premedication with dexamethasone based on the study due to the lack of information on the consequent effects on trastuzumab efficacy [25].

Another single institution article investigated the efficacy of additional premedications to reduce the risk of cetuximab-induced IRR in patients with head and neck SCC [26]. Touma et al. observed that 31.8% of their patients had an IRR using diphenhydramine alone [26]. The overall risk of IRRs was not significantly reduced with the addition of inhaled nebulised albuterol, famotidine or IV corticosteroids [26]. However, their premedication combination was effective in decreasing the specific risk of patients suffering from a high-grade IRR [26]. Akin to the anti-HER2 mAb recommendations, eviQ protocols also recommend dexamethasone premedication in all cycles of cetuximab therapy [27]. Notably, the Therapeutic Goods Association (TGA) PI and CMI state that patients “must receive premedication with an antihistamine and a corticosteroid at least 1 h prior to the administration of cetuximab” [28, 29].

The authors acknowledge that there are many clinical scenarios where ADCC-mediated mAbs are concurrently administered with chemotherapies that require corticosteroid premedication for other indications such as capillary leak syndrome [30].

## ADCC and steroids

To the authors’ knowledge, the first journal article linking corticosteroids with ADCC inhibition was published in 1978 by Cooper et al. who investigated why corticosteroids were contraindicated in the treatment of dendritic herpes keratitis. They demonstrated that this was not due to the steroids increasing the capacity of the cells to replicate type 1 herpes simplex virus (HSV) like originally thought, but rather inhibited human lymphocytes from mediating ADCC against HSV-infected fibroblasts [31•].

This was further investigated in 1984 by Nair et al. who examined the in vitro effect of prednisolone on NK cells and the ADCC activity of human lymphocytes [32]. It was found that prednisolone significantly supressed NK cell and ADCC activity and, of significant clinical relevance, that this was proportional to the concentration of the drug [32].

Expanding into the monoclonal antibody landscape, Kumai et al. have demonstrated that steroid treatment significantly inhibited cetuximab-induced NK cell ADCC activity [33••]. This was demonstrated in both head and neck SCC and CRC. It was additionally demonstrated that the expression of CD69, a marker for cytotoxic activity of NK cells induced by therapeutic antibodies, is decreased by dexamethasone stimulation [33••].

Sumikawa et al. have advocated for new chemotherapeutic regimens without glucocorticoid premedication due to their findings that dexamethasone interfered with trastuzumab-induced AKT suppression and subsequent pRB dephosphorylation and a breast cancer cell line [34••].

An interesting caveat to this discussion is the relationship between steroid premedication and CD20-targeted monoclonal antibodies for haematological malignancies. In trials investigating both rituximab and obinutuzumab, the addition of steroids improved ADCC [8, 35]. However, this is thought to be likely due to steroid-induced upregulation of CD20, the target of the aforementioned mAbs [36, 37].

## Conclusions

Corticosteroids are currently included in all cycles of monoclonal antibody administration as premedication as emesis prophylaxis, to avoid IRRs and combat syndromes associated with concurrent chemotherapy administration. However, upon review of the literature, IRRs are most likely to occur upon initial infusion; moreover, there are many alternatives for antiemesis with similar efficacy. Due to the proven reduction in anti-tumour activity of ADCC-mediated mAbs by corticosteroids, redundant use should be cautioned and clinicians should consider cessation of corticosteroids after the first administration unless clinically indicated. On a larger scale, the authors advocate for national guidelines and regulatory bodies such as the TGA to change their recommendations regarding steroid premedication in the setting of ADCC-mediated mAbs, acknowledging that there is a potential medicolegal risk due to the proven reduction in efficacy.
